# Pathophysiological Effects of Overactive STIM1 on Murine Muscle Function and Structure

**DOI:** 10.3390/cells10071730

**Published:** 2021-07-08

**Authors:** Roberto Silva-Rojas, Anne-Laure Charles, Sarah Djeddi, Bernard Geny, Jocelyn Laporte, Johann Böhm

**Affiliations:** 1 IGBMC (Institut de Génétique et de Biologie Moléculaire et Cellulaire), Inserm U1258, CNRS UMR7104, Université de Strasbourg, 67404 Illkirch, France; silvaror@igbmc.fr (R.S.-R.); djeddis@igbmc.fr (S.D.); 2 Fédération de Médecine Translationnelle de Strasbourg, Faculté de Médecine, Institut de Physiologie, Equipe d’Accueil UR3072 “Mitochondrie, Stress Oxydant et Protection Musculaire”, Université de Strasbourg, 67000 Strasbourg, France; anne.laure.charles@unistra.fr (A.-L.C.); bernard.geny@chru-strasbourg.fr (B.G.); 3 Service de Physiologie et d’Explorations Fonctionnelles, Pôle de Pathologie Thoracique, Nouvel Hôpital Civil, CHRU de Strasbourg, 67000 Strasbourg, France

**Keywords:** neuromuscular disorder, congenital myopathy, muscle weakness, York platelet syndrome, calcium, STIM2

## Abstract

Store-operated Ca^2+^ entry (SOCE) is a ubiquitous mechanism regulating extracellular Ca^2+^ entry to control a multitude of Ca^2+^-dependent signaling pathways and cellular processes. SOCE relies on the concerted activity of the reticular Ca^2+^ sensor STIM1 and the plasma membrane Ca^2+^ channel ORAI1, and dysfunctions of these key factors result in human pathologies. *STIM1* and *ORAI1* gain-of-function (GoF) mutations induce excessive Ca^2+^ influx through SOCE over-activation, and cause tubular aggregate myopathy (TAM) and Stormorken syndrome (STRMK), two overlapping disorders characterized by muscle weakness and additional multi-systemic signs affecting growth, platelets, spleen, skin, and intellectual abilities. In order to investigate the pathophysiological effect of overactive SOCE on muscle function and structure, we combined transcriptomics with morphological and functional studies on a TAM/STRMK mouse model. Muscles from *Stim1^R304W/+^* mice displayed aberrant expression profiles of genes implicated in Ca^2+^ handling and excitation-contraction coupling (ECC), and in vivo investigations evidenced delayed muscle contraction and relaxation kinetics. We also identified signs of reticular stress and abnormal mitochondrial activity, and histological and respirometric analyses on muscle samples revealed enhanced myofiber degeneration associated with reduced mitochondrial respiration. Taken together, we uncovered a molecular disease signature and deciphered the pathomechanism underlying the functional and structural muscle anomalies characterizing TAM/STRMK.

## 1. Introduction

Calcium (Ca^2+^) is a ubiquitous second messenger implicated in the regulation of fundamental adaptive and developmental processes in all cell types. The activation of Ca^2+^ pumps, Ca^2+^ exchangers, and Ca^2+^ channels in response to stimuli generates transient Ca^2+^ signals, which are decoded through transduction pathways to modulate transcription, induce cell growth and differentiation, and mediate nerve conduction, hormone release, coagulation, and muscle contraction [1]. Consistently, pathologic alterations of Ca^2+^ entry, Ca^2+^ storage, or Ca^2+^ release can severely impact Ca^2+^ signaling and disturb various molecular, physiological, and biochemical functions in the tissues and organs, resulting in human diseases [2].

Tubular aggregate myopathy (TAM) is a progressive muscle disorder caused by abnormal Ca^2+^ homeostasis and characterized by muscle weakness, myalgia, and cramps [3]. Most TAM patients also manifest a varying degree of additional multi-systemic signs such as thrombocytopenia, hyposplenism, miosis, ichthyosis, short stature, and dyslexia, and the full clinical picture constitutes the diagnosis of Stormorken syndrome (STRMK) [4,5,6,7]. TAM/STRMK arises from dominant gain-of-function (GoF) mutations in the Ca^2+^ sensor STIM1 and the Ca^2+^ channel ORAI1, and milder adult-onset cases with exclusive muscle involvement have been associated with mutations in the Ca^2+^ buffer calsequestrin (CASQ1) [8,9,10,11]. STIM1 and ORAI1 are key players of store-operated Ca^2+^ entry (SOCE), a ubiquitous mechanism triggering extracellular Ca^2+^ entry to refill the reticular Ca^2+^ stores and counteract the effects of Ca^2+^ deficit [12]. Functional investigations in the cellular model have shown that the *STIM1* and *ORAI1* mutations lead to excessive cytosolic Ca^2+^ levels through SOCE over-activation [9,11,13,14,15,16,17,18,19], and a recently reported TAM/STRMK mouse model harboring the most common STIM1 mutation p.Arg304Trp (R304W) was shown to exhibit elevated cytosolic Ca^2+^ levels in skeletal muscle and to recapitulate the main clinical signs of the human disorder including muscle weakness, thrombocytopenia, smaller size, and eye, skin, and spleen anomalies [20]. Histological and ultrastructural analyses of muscle sections from *Stim1^R304W/+^* mice confirmed the presence of fibers with Ca^2+^ overload, and additionally revealed increased muscle fiber degeneration and regeneration, as well as the presence of swollen mitochondria [20]. However, the precise molecular and cellular effect of overactive STIM1 and the associated Ca^2+^ excess on muscle function and structure remain elusive.

In order to determine the sequence of events leading to the muscle phenotype in *Stim1^R304W/+^* mice, we performed transcriptomic analyses on fast-twitch and slow-twitch muscles, and we identified major dysregulations of genes implicated in intracellular Ca^2+^ handling, excitation-contraction coupling (ECC), unfolded protein response (UPR), and mitochondrial dynamics. We performed complementary functional investigations on muscle contractibility and mitochondrial respiration, and we concluded that the STIM1-mediated elevated cytosolic Ca^2+^ levels interfere with muscle contraction and lead to sustained reticular stress, resulting in increased cell death and muscle fiber turnover, and thereby contribute to the muscle weakness and histological anomalies observed in TAM/STRMK.

## 2. Materials and Methods

### 2.1. Animal Care

Animal care and experimentation was in accordance with French and European legislation and approved by the institutional ethics committee (project numbers 2016031110589922 and 2020052817261437). Mice were housed in ventilated cages with free access to food and water in temperature-controlled rooms with 12 h day light/dark cycles. The *Stim1^R304W/+^* mouse line was described previously [20]. Sample size was determined based on Sigmastat sample size t-test and analysis of variance (ANOVA) calculator. All mice used in this study were 4 months old males as *Stim1^R304W/+^* mice are symptomatic at this age. They are smaller than their littermates, show spleen and eye movement defects, and manifest structural muscle anomalies [20].

### 2.2. DNA and RNA Studies

For DNA extraction, tibialis anterior and soleus muscle samples were homogenized in lysis buffer supplemented with 0.1 mg/mL protease K (Sigma-Aldrich, St. Louis, MO, USA) and incubated at 55 °C for 3 h. Following precipitation with 5M NaCl, DNA pellets were washed in ethanol and resuspended in H_2_O. Skeletal muscle RNA from tibialis anterior and soleus was extracted with TRI Reagent (Molecular Research Center, Cincinnati, OH, USA) and reverse transcribed using the SuperScript^TM^ IV Transcriptase (ThermoFisher Scientific, Waltham, MA, USA). For quantitative analyses, DNA and cDNA samples were amplified with the SYBR Green Master Mix I (Roche, Basel, Switzerland) on a LightCycler 480 Real-Time PCR System (Roche) using specific primer sets (Table S1). PCR products were Sanger-sequenced for validation.

For RNAseq, library preparation was performed with the TruSeq Stranded mRNA Sample Preparation Kit (Illumina, San Diego, CA, USA), and samples were single-end sequenced on a HiSeq4000 (Illumina). Raw data were preprocessed using cutadapt version 1.10 (https://doi.org/10.14806/ej.17.1.200), and reads with a Phred quality score above 20 and covering at least 40 nt were mapped onto the mouse genome mm10 assembly using STAR [21]. Gene expression was quantified using htseq-count [22] with annotations from Ensembl version 96 (http://www.ensembl.org/index.html) and union mode, and normalized with DESeq2 [23]. For the establishment of sample-to-sample distances heatmaps, hierarchical clustering was performed using the UPGMA (unweighted pair group method with arithmetic mean) algorithm. Gene ontology analyses were performed with ClusterProfiler [24] using the overrepresentation test and the Benjamini–Hochberg correction for multiple testing. Enrichments with a corrected *p*-value < 0.05 were considered significant.

### 2.3. Protein Studies

For western blot, tibialis anterior and soleus muscles were homogenized in RIPA (radio immunoprecipitation) buffer supplemented with 1 mM PMSF and complete mini EDTA-free protease inhibitor cocktail (Roche). Denatured protein samples were loaded on SDS-PAGE, and transferred to a nitrocellulose membrane using the Transblot^®^ TurboTM RTA Transfer Kit (Bio-Rad, Hercules, CA, USA). The following primary and secondary antibodies were used: mouse anti-DHPR (sc-514685, Santa Cruz Biotechnology, Dallas, TX, USA), mouse anti-RyR1 (MA3-925, ThermoFisher Scientific, Waltham, MA, USA), mouse anti-SERCA1 (MA3-911, ThermoFisher Scientific, Waltham, MA, USA), mouse anti-PGC1α (AB3242, Merck Millipore, Burlington, MA, USA), mouse anti-OXPHOS (ab110413, Abcam, Cambridge, UK), rabbit anti-LC3 (NB100-2220, Novus Biologicals, Littleton, CO, USA), mouse anti-P62 (H00008878-M01, Abnova, Taipeh, Taiwan), peroxidase-coupled goat anti-rabbit (112-036-045, Jackson ImmunoResearch, Ely, UK), and peroxidase-coupled goat anti-mouse (115-036-068, Jackson ImmunoResearch, Ely, UK). Immunoblots were revealed with the Supersignal west pico kit (ThermoFisher Scientific, Waltham, MA, USA), and monitored on the Amersham Imager 600 (GE Healthcare Life Sciences, Chicago, IL, USA). Ponceau S staining (Sigma-Aldrich, St. Louis, MO, USA) served as loading control.

For immunohistochemistry, 8 µm muscle sections were incubated with the following antibodies: mouse anti-myosin heavy chain type I (BA-D5, DSHB, Iowa City, IA, USA), mouse anti-myosin heavy chain type IIa (SC-71, DSHB, Iowa City, IA, USA), mouse anti-myosin heavy chain type IIb (BF-F3, DSHB, Iowa City, IA, USA), homemade rabbit anti-cleaved caspase-3, mouse anti-embryonic myosin heavy chain (F1.652, DSHB, Iowa City, IA, USA), Cy3-coupled goat anti-mouse (115-165-207, Jackson ImmunoResearch, West Grove, PA, USA), Cy5-coupled goat anti-mouse (115-545-205, Jackson ImmunoResearch, West Grove, PA, USA), Dylight^TM^ 405-coupled goat anti-mouse (115-475-075, Jackson ImmunoResearch, West Grove, PA, USA), and Alexa Fluor^TM^ 555-coupled goat anti-rabbit (A21430, ThermoFisher Scientific, Waltham, MA, USA). The sarcolemma was stained with Wheat Germ Agglutinin, Alexa Fluor^TM^ 647 conjugate (ThermoFisher Scientific, Waltham, MA, USA). Images were recorded with the Nanozoomer 2HT slide scanner (Hamamatsu Photonics, Hamamatsu, Japan), fiber type percentage was assessed using MuscleJ plug-in [25], and the percentage of regenerating fibers was assessed with cell counter plug-in.

### 2.4. In Situ Muscle Contraction Measurements

Mice were anesthetized through intraperitoneal injection of a domitor/fentanyl mix (2/0.28 mg/Kg), diazepam (8 mg/Kg), and fentanyl (0.28 mg/Kg). The distal tibialis anterior tendons were excised and attached to the Complete1300A isometric transducer (Aurora Scientific, Aurora, ON, Canada), and the sciatic nerve was stimulated by a single pulse of 1 Hz. Muscle contraction and relaxation speed reflect the time between stimulation and maximal force production, and the time until force decreases by 50%.

### 2.5. Mitochondrial Respiration

Tibialis anterior and soleus were dissected from anesthetized mice, kept in Krebs-HEPES buffer for preparation, and permeabilized by incubation in buffer S containing saponin (50 µg/mL) as previously described [26,27]. The samples were then placed into the Oxygraph-2k chamber (Oroboros instruments, Innsbruck, Austria) containing buffer R+BSA and a Clark electrode to analyze non-phosphorylating respiration and oxidative phosphorylation using a multiple substrate-uncoupler-inhibitor titration (SUIT) protocol. Complex I-linked substrate state was measured at 37 °C under continuous stirring following the addition of glutamate and malate. Then, ADP was added to activate oxidative phosphorylation through complex I (CI-linked OXPHOS state), and succinate to activate complex II (CI&II-linked OXPHOS state). Oxygen consumption is expressed as pmol/(s*mg) wet weight. H_2_O_2_ production was assessed simultaneously by adding amplex red and HRP in the Oxygraph-2k chamber, and is expressed as nmol/(s*mg) wet weight.

To measure superoxide anion, muscle fragments were incubated for 30 min in Krebs-HEPES buffer containing DETC and deferoxamin in a thermo-regulated incubator at 37 °C under gas mix (O2: 2.7%, N2: 97.8%) and controlled pressure (20 mmHg; Gas Treatment Chamber BIO-V and Temperature & Gas Controller BIO-III, Noxygen^®^, Elzach, Germany). Samples were put on ice, and oxidized probe concentrations were measured using the e-scan spectrometer (Bruker Win-EPR^®^, Elzach, Germany). Finally, muscle fragments were dried for 15 min at 150 °C. The results are expressed in µmol/(min*mg) dry weight.

### 2.6. Statistical Analyses

All experiments were performed and analyzed in a blinded manner and the investigators were unaware of the genotype of the mice. Data were verified for normal distribution using the Shapiro–Wilk test, and are presented as mean ± SEM. For normally distributed data, statistical differences between wild-type (WT) and *Stim1^R304W/+^* mice were examined using the Student’s t-test (with or without Welch’s correction). For other data, a non-parametric Mann–Whitney statistical test was performed.

## 3. Results

### 3.1. Transcriptomics Identifies Dysregulated Molecular Networks in Stim^R304W/+^ Tibialis Anterior

*Stim1^R304W/+^* mice were previously shown to exhibit abnormal muscle contraction properties, and morphological analyses of muscle sections revealed fiber atrophy and the presence of internalized nuclei, indicating muscle fiber degeneration [20]. This was further supported by an elevated expression of myogenic differentiation markers in muscle samples and by increased serum creatine kinase levels in the blood of the animals [20].

To shed light on the molecular pathways affected by overactive STIM1 and to decipher the sequence of events leading to the muscle phenotype, we generated gene expression profiles through RNAseq on tibialis anterior muscle extracts from *Stim1^R304W/+^* mice and WT littermates.

**Figure 1:**
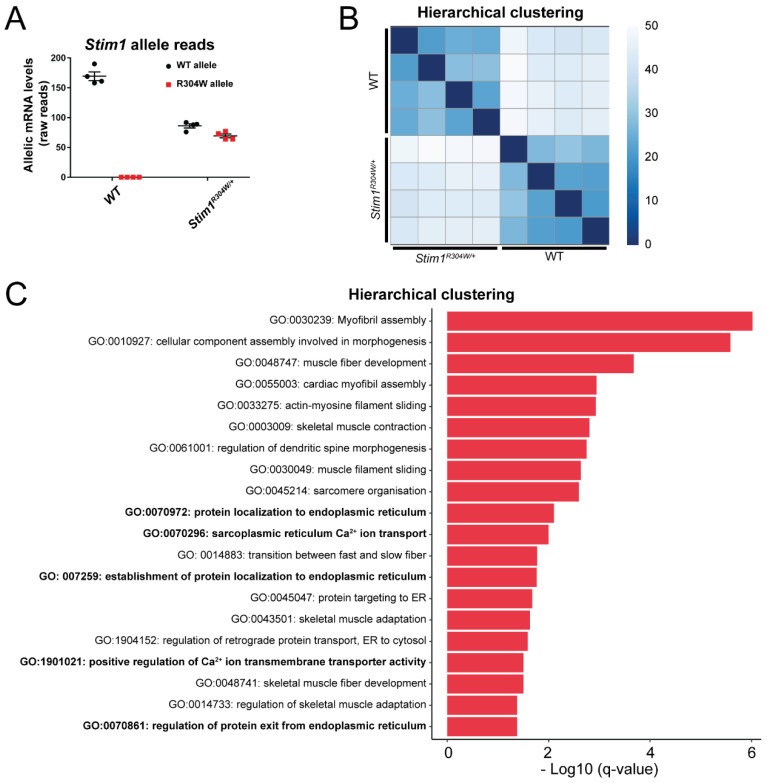
Transcriptomic profile of *Stim1^R304W/+^* muscle. (**A**) RNAseq on *Stim1^R304W/+^* tibialis anterior samples revealed a comparable expression of the mutant and WT *Stim1* alleles (*n* = 4). (**B**) Hierarchical clustering of the RNAseq data evidenced distinct sample grouping of WT and *Stim1^R304W/+^* mice (*n* = 4). (**C**) Classification of abnormally expressed genes in *Stim1^R304W/+^* tibialis anterior into gene ontology (GO) terms revealed an enrichment of groups associated with myofibril assembly and morphogenesis, Ca^2+^ transport and sarcoplasmic reticulum, and sarcomere organization and contraction. GO categories related to the immune system were removed from the analysis.

Both *Stim1* alleles were expressed at comparable levels in *Stim1^R304W/+^* muscle (Figure 1A), and hierarchical clustering of the data resulted in separate sample grouping of the *Stim1^R304W/+^* and WT transcriptomes (Figure 1B). We detected a total of 3346 differentially expressed genes, which were classified into subcategories based on gene ontology (GO) terms. Several groups including the largest category GO:0002274 (myeloid leukocyte activation) were, however, unrelated to skeletal muscle and essentially encompassed genes associated with immune response (Figure S1A). This was expected because myofiber degeneration involves fiber clearance, which is mediated by immune cells [28]. We removed all groups falling under the parental GO:0002376 term immune system process (Figure S1B), and the remaining 2841 differentially expressed genes divided into GO categories associated with myofibril assembly and morphogenesis, Ca^2+^ transport and sarcoplasmic reticulum, or sarcomere organization and contraction, all reflecting essential processes in skeletal muscle development and physiology (Figure 1C).

### 3.2. Altered Regulators of Ca^2+^ Handling and Excitation-Contraction Coupling in Stim1^R304W/+^ Tibialis Anterior

In accordance with the assumption that TAM/STRMK is mainly caused by excessive extracellular Ca^2+^ influx [9,11], genes implicated in the Ca^2+^ transport across the sarcolemma (GO:1901021) and the sarcoplasmic reticulum membrane (GO:0070296) were considerably dysregulated in *Stim1^R304W/+^* muscle. In order to validate the RNAseq data, we determined the relative expression of selected genes and proteins driving intracellular Ca^2+^ handling and Ca^2+^-related excitation-contraction coupling (ECC) by RT-qPCR and western blot.

**Figure 2:**
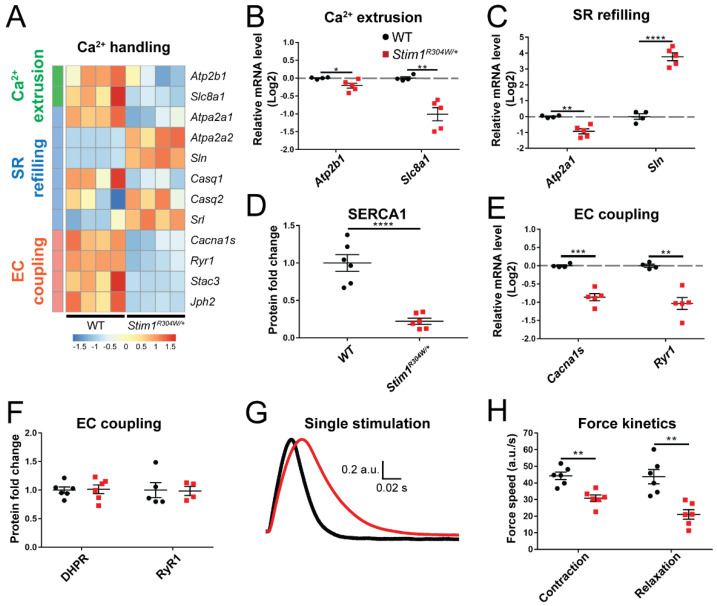
Altered expression of Ca^2+^ handling genes in *Stim1^R304W/+^* muscle and abnormal contraction and relaxation properties. (**A**) RNAseq heatmap illustrating the relative expression of genes implicated in Ca^2+^ extrusion, sarcoplasmic reticulum (SR) refilling, and excitation-contraction (EC) coupling in *Stim1^R304W/+^* and wild-type (WT) tibialis anterior (*n* = 4). (**B**) RT-qPCR showing reduced expression of *Atp2b1* (encoding a plasma membrane Ca^2+^ pump) and *Slc8a1* (plasma membrane Na^+^/Ca^2+^ exchanger) in *Stim1^R304W/+^* mice (*n* = 4–5). (**C**,**D**) SERCA1 gene expression and protein levels are reduced in *Stim1^R304W/+^* mice, while *Sln* expression is increased (*n* = 4–6). (**E**,**F**) Downregulation of *Cacna1s* and *Ryr1* in *Stim1^R304W/+^* tibialis anterior, but normal DHPR and RyR1 protein levels (*n* = 4–6). (**G**,**H**) Mean normalized force production following a single 1 Hz stimulation showing delayed muscle contraction and relaxation speed in *Stim1^R304W/+^* mice (*n* = 6). Significant differences are illustrated as * (*p* < 0.05), ** (*p* < 0.01), *** (*p* < 0.001), and **** (*p* < 0.0001).

Each cell possesses a panel of Ca^2+^ channels, Ca^2+^ pumps, and Ca^2+^ exchangers to control Ca^2+^ flows within the cytosol and between the organelles and orchestrate the complex spatiotemporal interplay of Ca^2+^-dependent pathways and processes. *Atp2b1* and *Slc8a1*, encoding a plasma membrane Ca^2+^ pump and a Na^+^/Ca^2+^ exchanger, respectively, were significantly downregulated in *Stim1^R304W/+^* muscles compared with controls (Figure 2A,B). This points to an impaired extrusion of excessive Ca^2+^ from the cytosol, and provides an explanation for the permanently elevated cytosolic Ca^2+^ levels in TAM/STRMK muscle fibers. In agreement, we also measured a decreased expression of *Atp2a1*, encoding the reticular Ca^2+^ pump SERCA1, and a simultaneous upregulation of *Sln*, coding for the SERCA1 inhibitor sarcolipin in the *Stim1^R304W/+^* muscles (Figure 2A,C,D and Figure S2A).

ECC refers to the generation of muscle force through a multistep process beginning with the electrical stimulation of the voltage-gated Ca^2+^ channel DHPR at the plasma membrane, and concluding with the activation of the reticular Ca^2+^ channel RyR1 and the subsequent release of Ca^2+^ to the cytosol [29]. Cytosolic Ca^2+^ overload is known to interfere with ECC [30,31], and consistently, we observed a reduced expression of *Cacna1s*, encoding the pore-forming subunit of DHPR, and of *Ryr1* and the ECC-regulating genes *Stac3* and *Jph2* in *Stim1^R304W/+^* muscle samples (Figure 2A,E). We, however, also noted that the DHPR and RyR1 protein levels were similar in TAM/STRMK and WT mice (Figure 2F and Figure S2B,C). As the RT-qPCR and western blot data were not fully conclusive, we investigated the in situ muscle contraction properties of *Stim1^R304W/+^* and WT tibialis anterior to determine a possible functional alteration of ECC. Following single-pulse stimulations of the sciatic nerve, *Stim1^R304W/+^* mice manifested a delay in muscle force production compared with the controls (Figure 2G,H and Figure S2D), reflecting a defective coupling between excitation and contraction. We also observed a delay in muscle relaxation in *Stim1^R304W/+^* muscles (Figure 2G,H), supposedly resulting from the abundance of Ca^2+^ in the cytosol and at the contractile unit.

Taken together, our data suggest that the Ca^2+^ extrusion systems in *Stim1^R304W/+^* muscle fibers are impaired and enhance the cytosolic Ca^2+^ surcharge induced by SOCE overactivity, compromising both muscle contraction and relaxation kinetics.

### 3.3. Less Mitochondria in Stim1^R304W/+^ Tibialis Anterior

**Figure 3:**
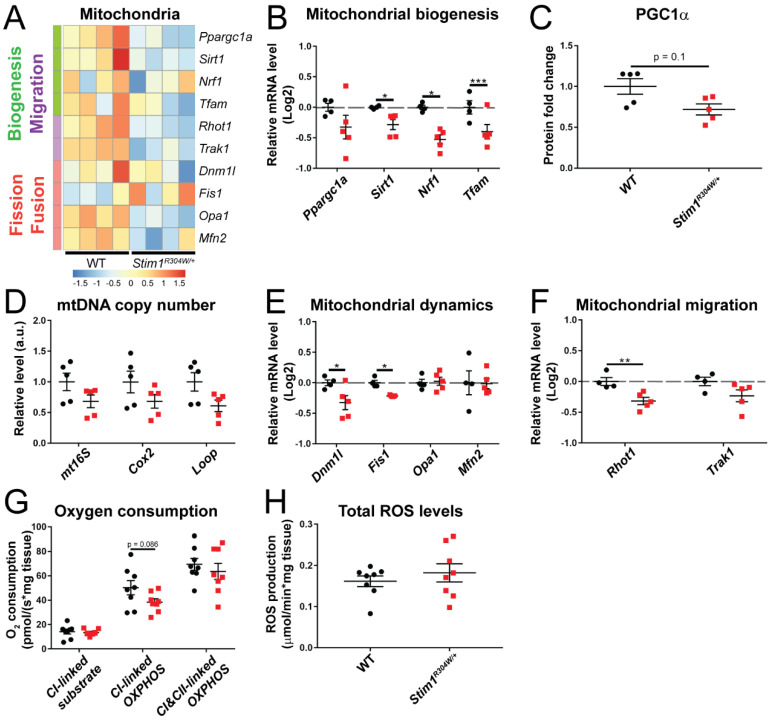
Minor mitochondrial defects in *Stim1^R304W/+^* muscle. (**A**) RNAseq heatmap showing the relative gene expression of genes involved in mitochondrial biogenesis, migration, fission, and fusion in *Stim1^R304W/+^* and WT tibialis anterior (*n* = 4). (**B**,**C**) RT-qPCR confirmed the reduction of the mitochondrial biogenesis genes *Sirt1*, *Nrf1*, and *Tfam* in *Stim1^R304W/+^* mice, and indicates a decreased PGC1α (*Ppargc1a*) gene expression and protein level (*n* = 4–5). (**D**) The quantities of the mitochondrial copy number maker genes *mt16S*, *Cox2*, and *Loop* tend to be reduced in *Stim1^R304W/+^* tibialis anterior (*n* = 5). (**E**,**F**) Mitochondrial fission (*Dnm1l* and *Fis1*) and migration (*Rhot1*) genes are downregulated in *Stim1^R304W/+^* mice (*n* = 4–5). (**G**,**H**) Analysis of oxygen consumption shows a comparable mitochondrial non-phosphorylating respiration (CI-linked substrate), oxidative phosphorylation (CI-linked OXPHOS and CI/CII-linked OXPHOS), and ROS production in *Stim1^R304W/+^* and WT tibialis anterior (*n* = 8). Significant differences are illustrated as * (*p* < 0.05), ** (*p* < 0.01), and *** (*p* < 0.001).

Mitochondria were the first intracellular organelles to be associated with an active role in Ca^2+^ homeostasis [32]. They act as Ca^2+^ buffers to rapidly remove Ca^2+^ from the cytosol, and can free large amounts of Ca^2+^ in defined subcellular domains to generate local Ca^2+^ gradients [33]. It is, therefore, possible that the constitutive Ca^2+^ excess in *Stim1^R304W/+^* muscle fibers overcharges the mitochondria and accounts for the mitochondrial swelling observed by electron microscopy [20]. Comparative analysis of the RNAseq, RT-qPCR, and western blot data revealed a moderately reduced expression of genes implicated in mitochondrial biogenesis (*Ppargc1a*, *Sirt1*, *Nrf1*, *Tfam*) and decreased levels of proteins of the electron transport chain, suggesting a lower number of mitochondria in *Stim1^R304W/+^* tibialis anterior samples compared with controls (Figure 3A–C and Figure S3A–C). Indeed, quantification of the *mt16S*, *Cox2*, and *Loop* genes, all encoded on the mitochondrial DNA, confirmed a tendency towards a reduced mitochondrial copy number in *Stim1^R304W/+^* muscle (Figure 3D). We furthermore found a downregulation of genes and proteins driving mitochondrial migration (*Rhot1* and *Trak1*) and fission (*Dnm1l*, *Fis1*), while the expression of genes relevant for mitochondrial fusion (*Opa1* and *Mfn2*) was comparable in *Stim1^R304W/+^* and WT mice (Figure 3A,E,F). To explore a potential impact of the molecular defects on organelle function, we next determined mitochondrial respiration in dissected *Stim1^R304W/+^* and WT tibialis anterior muscles.

Respirometric and spectrometric analyses revealed a slight, but not significant reduction of complex I-linked substrate state, complex I-linked OXPHOS state, and complex I/complex II- linked OXPHOS state in *Stim1^R304W/+^* muscle, and a comparable reactive oxygen species (ROS) production in *Stim1^R304W/+^* mice and controls (Figure 3G,H and Figure S3D). Overall, our findings suggest that mitochondrial respiration is largely normal in *Stim1^R304W/+^* muscles. We, however, found evidence of a decreased mitochondrial number, which may contribute to the muscle weakness in TAM/STRMK mice and patients. The swollen mitochondria possibly results from impaired mitochondrial fission.

### 3.4. ER Stress and Increased Cell Death in Stim1^R304W/+^ Tibialis Anterior

**Figure 4:**
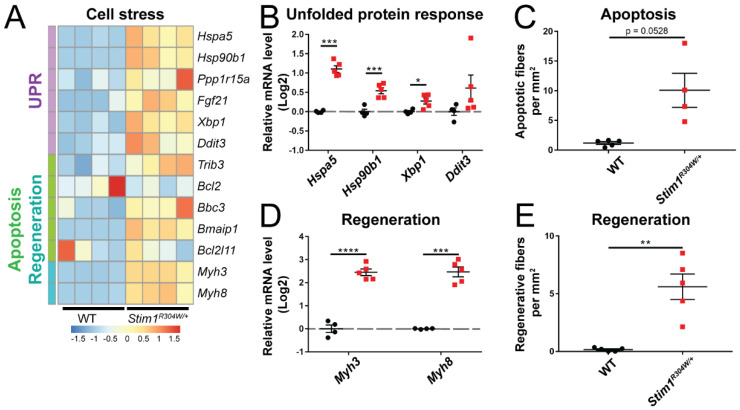
Unsolved reticular stress leading to muscle fiber turnover in *Stim1^R304W/+^* mice. (**A**) RNAseq heatmap depicting the relative gene expression of genes implicated in unfolded protein response (UPR), apoptosis, and muscle fiber regeneration in *Stim1^R304W/+^* and WT tibialis anterior (*n* = 4). (**B**) RT-qPCR validates the upregulation of genes encoding chaperones (*Hspa5* and *Hsp90b1*), and the XBP1 transcription factor (*Xbp1*) in *Stim1^R304W/+^* tibialis anterior (*n* = 4–5). (**C**) Apoptotic fibers tend to be more abundant on *Stim1^R304W/+^* muscle sections than in controls (*n* = 4–5). (**D**,**E**) Upregulation of the embryonic (*Myh3*) and perinatal (*Myh8*) myosin genes in *Stim1^R304W/+^* tibialis anterior, and significant increase of regenerating muscle fibers (*n* = 4–5). Significant differences are illustrated as * (*p* < 0.05), ** (*p* < 0.01), *** (*p* < 0.001), and **** (*p* < 0.0001).

The sarcoplasmic reticulum (SR) is a specialized type of smooth endoplasmic reticulum (ER), represents the primary Ca^2+^ storage organelle in striated muscle cells, and controls intracellular Ca^2+^ cycling through the concerted regulation of Ca^2+^ uptake, Ca^2+^ storage, and Ca^2+^ release [34]. The dysregulation of Ca^2+^ homeostasis in the ER/SR promotes the accumulation of unfolded or misfolded proteins and initiates a protective process known as UPR (unfolded protein response), which interrupts protein translation, degrades unfolded proteins, and activates signaling pathways to produce chaperones [35]. Numerous UPR genes (*Hspa5*, *Hsp90b1*, *Xbp1*, *Ddit3*) were overexpressed in *Stim1^R304W/+^* muscles compared with WT controls, indicating an important Ca^2+^ stress (Figure 4A,B). We also noticed an upregulation of genes associated with apoptosis (*Bbc3*, *Bmaip1*, *Trib3*), and immunofluorescence experiments confirmed the higher incidence of apoptotic fibers on *Stim1^R304W/+^* muscle sections (Figure 4A,C and Figure S4). This is in agreement with the notion that continuous Ca^2+^ stress ultimately leads to cell death [35]. Along with the histological signs of muscle fiber degeneration [20], the upregulation of genes involved in muscle fiber regeneration (*Myh3*, *Myh8*), and the occurrence of regenerating fibers in *Stim1^R304W/+^* muscle (Figure 4A,D,E and Figure S4), our data suggest that the STIM1 R304W mutation induces constitutive ER/SR stress and results in increased cycles of muscle fiber degeneration and regeneration.

### 3.5. Comparison between Stim1^R304W/+^ Fast-Twitch and Slow-Twitch Muscles

Skeletal muscle is composed of slow-twitch type I and fast-twitch type II fibers, and the ratio and distribution of the individual fiber types characterizes each muscle and its adaptation to either powerful movements or endurance activities [36,37]. The glycolytic tibialis anterior muscle, essentially containing fast type II muscle fibers, was used for the quantification of gene expression and the consecutive functional investigations on *Stim1^R304W/+^* and WT mice. In order to provide a comparative analysis between fast-twitch and slow-twitch muscles and to explore a potentially diverging effect of overactive STIM1 on slow type I fibers, we assessed the relative expression of selected marker genes in soleus muscle extracts from *Stim1^R304W/+^* mice and controls. This is of particular interest as type I and type II muscle fibers differ in their SR Ca^2+^ content and cytosolic Ca^2+^ concentration at rest, and feature a different Ca^2+^ sensitivity [38,39].

**Figure 5:**
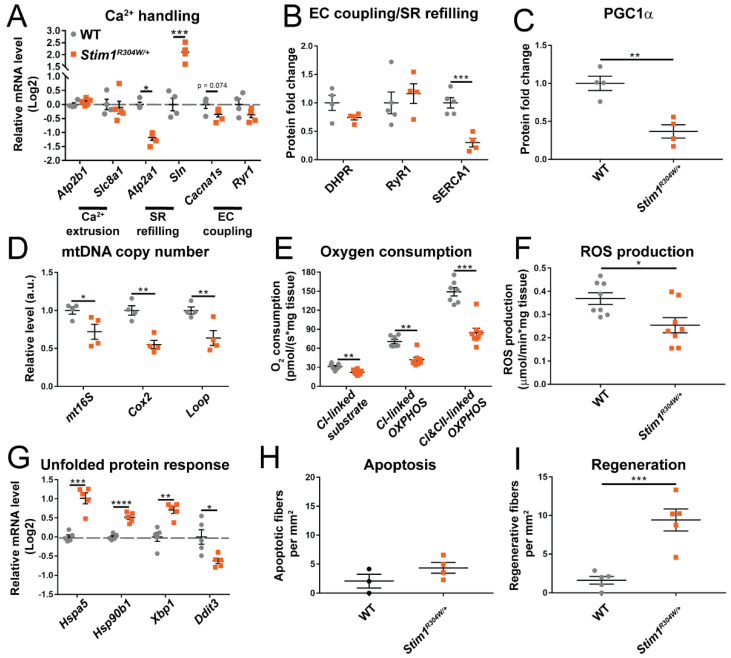
Defective Ca^2+^ handling, reduced mitochondrial copy number, and increased reticular stress in *Stim1^R304W/+^* slow-twitch muscle. (**A**,**B**) RT-qPCR on selected genes illustrates a reduction of SERCA1 gene expression and protein level and a simultaneous upregulation of the SERCA1 inhibitor *Sln* in *Stim1^R304W/+^* soleus, while other genes involved in Ca^2+^ handling are normally expressed (*n* = 4–5). (**C**,**D**) In agreement with the reduced protein level of the mitochondrial biogenesis regulator PGC1α in *Stim1^R304W/+^* soleus, the mitochondrial copy number marker genes *mt16s*, *Cox2*, and *Loop* are decreased compared with the WT (*n* = 4). (**E**,**F**) Oxygen consumption and ROS production are significantly lower in *Stim1^R304W/+^* compared with WT soleus (*n* = 7–8). (**G**) Increased UPR in *Stim1^R304W/+^* soleus as illustrated by the upregulation of the stress-regulated genes *Hspa5*, *Hsp90b1*, and *Xbp1* (*n* = 4–5). (**H**,**I**) *Stim1^R304W/+^* soleus sections show a tendency of augmented apoptosis and a significant increase of regenerating fibers compared with controls (*n* = 3-5). Significant differences are illustrated as * (*p* < 0.05), ** (*p* < 0.01), *** (*p* < 0.001), and **** (*p* < 0.0001).

In analogy to the fast-twitch tibialis anterior, RT-qPCR uncovered a downregulation of *Serca1* and a simultaneous upregulation of the SERCA1 inhibitor *Sln* in the slow-twitch soleus muscle (Figure 5A,B and Figure S5A–C). We, however, noted a normal expression level of *Atp2b1* and *Slc8a1* (Figure 5A), indicating that the extrusion of excessive Ca^2+^ may be less affected in the soleus. Compared with tibialis anterior, the mitochondrial copy number was markedly reduced in *Stim1^R304W/+^* soleus, and this was substantiated by a significant decrease in mitochondrial non-phosphorylating respiration, oxidative phosphorylation, and ROS production (Figure 5C–F and Figure S6A–D). As type I and type II muscle fibers differ in mitochondrial content and mitochondrial activity, we next explored the fiber type composition in *Stim1^R304W/+^* soleus. Immunofluorescence experiments on transverse muscle sections evidenced an increased ratio of mitochondria-rich type I muscle fibers in *Stim1^R304W/+^* mice compared with the WT (Figure S7A), suggesting a fast-to-slow muscle fiber conversion associated with relevant mitochondrial loss in *Stim1^R304W/+^* soleus, and highlighting a fiber type-specific effect of the STIM1 R304W mutation on mitochondria. Finally, the marker genes for ER/SR stress and myofiber regeneration were overexpressed in the *Stim1^R304W/+^* soleus compared with the control, and immunofluorescence on muscle sections provided the evidence for increased apoptosis and muscle fiber regeneration as observed in tibialis anterior (Figure 5G–I and Figure S7B).

In summary, *Stim1^R304W/+^* tibialis anterior and soleus displayed comparable molecular defects of ECC, reticular Ca^2+^ uptake, and ER physiology, while the deficits in mitochondrial copy number and respiration were more pronounced in the soleus. Apart from the differences in mitochondrial content and activity, our data indicate that the aberrant Ca^2+^ homeostasis in TAM/STRMK affects slow-twitch and fast-twitch muscle fibers to a similar extent, leading to muscle fiber degeneration of both type I and type II fibers.

## 4. Discussion

### 4.1. From Constitutive STIM1 Activation to Abnormal Muscle Contraction and Relaxation Kinetics, Muscle Fiber Degeneration, and Mitochondrial Loss

Tubular aggregate myopathy (TAM) and Stormorken syndrome (STRMK) are overlapping disorders principally resulting from abnormal Ca^2+^ balance and affecting skeletal muscle, platelets, spleen, and skin. *Stim1^R304W/+^* mice expressing a constitutively active STIM1 mutant feature cytosolic Ca^2+^ overload in the muscle fibers and manifest functional and structural muscle anomalies [20]. Here, we deciphered the sequence of events triggered by overactive STIM1 and leading to the muscle phenotype in the TAM/STRMK mouse model, and we provide transcriptomic, proteomic, and functional data. We demonstrate that the STIM1-mediated abundance of Ca^2+^ impedes accurate muscle contraction and relaxation of tibialis anterior, and induces constitutive reticular stress in both slow-twitch and fast-twitch muscles, ultimately leading to myofiber degeneration and mitochondrial loss.

The coordinated process of muscle contraction is intrinsically linked to the strict regulation of the Ca^2+^ flows between the sarcoplasmic reticulum and the cytosol, hosting the contractile unit. Ca^2+^ triggers the shortening of the sarcomere to generate muscle force, and muscle relaxation occurs when Ca^2+^ is pumped back to the SR [40]. The dysregulation of Ca^2+^ homeostasis can thus interfere with proper excitation-contraction coupling and compromise normal muscle function. In line with the elevated resting Ca^2+^ levels in the cytosol of *Stim1^R304W/+^* myotubes [20], our in situ force measurement on the murine TAM/STRMK model disclosed extensive ECC perturbations as shown by the aberrant muscle contraction kinetics. The delayed relaxation of *Stim1^R304W/+^* tibialis anterior following muscle contraction most probably reflects a direct consequence of the inefficient Ca^2+^ removal from the sarcomere, and presumably accounts for the muscle stiffness, cramps, and myalgia observed in TAM/STRMK patients [5]. The Ca^2+^ abundance at the contractile unit primarily comes from the excessive extracellular Ca^2+^ influx through SOCE over-activation, and is exacerbated by the downregulation of the SR Ca^2+^ pump SERCA1 and the reduced expression of plasma membrane Ca^2+^ pumps and Ca^2+^ exchangers, resulting in the inability of the muscle fibers to efficiently clear the Ca^2+^ from the cytosol.

The SERCA1 downregulation and the concurrent upregulation of the SERCA1 inhibitor sarcolipin in both tibialis anterior and soleus possibly represent a protective effort of the *Stim1^R304W/+^* muscle fibers to limit reticular Ca^2+^ overload and ensure ordered protein synthesis, folding, modification, and transport. Our RNAseq data, however, uncovered a significant overexpression of several UPR marker genes, evidencing distinct reticular Ca^2+^ stress in *Stim1^R304W/+^* muscle. In accordance with the fact that steady reticular stress ineluctably leads to cell death [35], we detected signs of apoptosis and enhanced muscle fiber degeneration and regeneration cycles in *Stim1^R304W/+^* mice. Muscle fiber degeneration also involved mitochondrial loss especially in the soleus, which is principally composed of mitochondria-rich type I muscle fibers. Noteworthy, we found an increased ratio of type I fibers in the soleus from *Stim1^R304W/+^* mice, indicating a conversion from fast to slow myofibers. This is in accordance with previous findings in mice carrying a *Cacna1s* mutation and displaying elevated cytosolic Ca^2+^ levels [41], and a shift towards slow-twitch muscle fibers was also observed in rabbits following muscle fiber degeneration and regeneration [42]. This suggests that the altered myofiber composition in *Stim1^R304W/+^* soleus is a direct consequence of the Ca^2+^-induced muscle fiber degeneration.

### 4.2. Physiological and Structural Similarities in TAM/STRMK and Other Disorders Affecting ECC

Abnormal Ca^2+^ homeostasis interfering with regular muscle contraction and impacting efficient force production is also seen in disorders affecting the key players of the ECC machinery. A large number of mutations in *RYR1* are associated with central core disease (CCD), clinically characterized by childhood-onset hypotonia and proximal muscle weakness [43]. Functional investigations have shown that the mutations either alter the interaction with DHPR, or generate a leaky RyR1 Ca^2+^ channel involving a constitutive cytosolic Ca^2+^ overload [44,45,46]. In any case, the amount of released Ca^2+^ upon membrane depolarization and DHPR activation is reduced, evidencing an uncoupling of excitation from contraction [47]. In analogy and reflecting significant ECC defects, the *Stim1^R304W/+^* mice manifested a delay in muscle force production and a downregulation of *RyR1* and *Cacna1s*. Reduced *RYR1* expression levels were also found in differentiated myotubes derived from a TAM/STRMK patient carrying the STIM1 p.Leu96Val (L96V) mutation and presenting with early-onset lower limb muscle weakness and myalgia [48,49], emphasizing the importance of normal cellular Ca^2+^ balance for effective muscle contraction, and suggesting that the Ca^2+^-related dysregulation of ECC contributes to the muscle weakness characterizing CCD and TAM/STRMK.

Other *RYR1* mutations render the RyR1 channel hypersensitive to triggering agents in volatile anesthetics and induce excessive Ca^2+^ release from the SR, resulting in malignant hyperthermia (MH) [50,51]. MH is a potentially lethal disorder involving uncontrolled contractures, hyperkalemia, hypermetabolism, and cardiac arrhythmia [52,53]. In a similar way, specific mutations in *CACNA1S*, encoding the alpha-1S subunit of the voltage-gated Ca^2+^ channel DHPR, were shown to increase the sensitivity of RyR1 to activation, resulting in elevated resting Ca^2+^ levels in the cytosol [54,55]. It is noteworthy that muscle biopsies from MH patients display alterations of mitochondrial shape and distribution, indicating abnormal mitochondrial dynamics [56]. This is comparable to the ultrastructural pictures of the mitochondria in muscle samples from the *Stim1^R304W/+^* mice and another TAM/STRMK mouse model carrying the STIM1 I115F mutation [57], and conforms to our RNAseq and RT-qPCR data indicating a reduced expression of genes implicated in mitochondrial fission and migration. Taking into account that cytosolic Ca^2+^ overload is a hallmark of MH and TAM/STRMK, it is conceivable that both disorders share at least partially a common pathomechanism. Although not reported in the literature, TAM/STRMK patients may thus be at risk for MH, and this is supported by the fact that mice lacking the TAM gene *Casq1* exhibit a MH-like phenotype [58]. However, our respirometric experiments provided evidence that mitochondrial respiration is decreased, but functionally normal in *Stim1^R304W/+^* muscle, suggesting that mitochondrial dysfunction is not a contributing factor for TAM/STRMK. In accordance, *Casq1* null mice did not show anomalies of the mitochondrial morphology [58]. The reduced mitochondrial copy number, especially in *Stim1^R304W/+^* slow-twitch muscle fibers, might nevertheless provoke a gap in the required energy production and add to the muscle weakness in TAM/STRMK mice and patients.

### 4.3. Ca^2+^ Stress in Myopathies and Dystrophies, and Potential Treatment Options

Mice carrying the CCD-related RyR1 mutation p.Ile4895Thr (I4895T) in the pore-forming C-terminus of the channel were reported to manifest increased reticular stress, leading to the activation of UPR in muscle fibers [59], highlighting yet another similarity between *RYR1*-related disorders and TAM/STRMK. This congruence can be explained by the mechanistic interconnection between ECC and SOCE. Indeed, calsequestrin (CASQ1) actively regulates the amount of Ca^2+^ release from the SR in a quaternary complex with RyR1, junction, and triadin [60], and sequesters STIM1 upon Ca^2+^ store depletion, thereby acting as a negative regulator of SOCE [61]. STIM1 was furthermore found to bind DHPR and to suppress depolarization-induced channel opening [62], illustrating a reciprocal regulation of ECC and SOCE.

Of note, treatment of the *Ryr1^I4895T/+^* mice with the chemical chaperone 4-PBA reduced reticular stress and improved skeletal muscle function [59], suggesting that the anticipation of UPR may have a similar therapeutic effect for TAM/STRMK mice and prevent myofiber degeneration. UPR along with a high cytosolic Ca^2+^ content and aberrant ECC was also described in Duchenne muscular dystrophy (DMD), associating progressive muscle loss with dilated cardiomyopathy [63,64], and administration of 4-PBA reduced exercise-induced muscle damage and considerably improved the muscle phenotype in mdx mice, a well-studied murine model for DMD [65]. Moreover, treatment with the ECC effector taurine, overexpression of SERCA, or silencing of the SERCA inhibitor Sarcolipin efficiently decreased the cytosolic Ca^2+^ levels, restored ECC, and alleviated muscle fiber degeneration in mdx mice [66,67,68], potentially representing additional therapeutic options for TAM/STRMK. Several TAM/STRMK mouse models carrying different STIM1 mutations as D84G [69], I115F [57], or R304W [20,70] exist, and the animals diverge in the occurrence and severity of the muscle and multi-systemic signs. These models thus represent valuable tools to establish general or mutation-specific treatments and validate their potency to attenuate or revert the muscle, spleen, skin, or platelet phenotypes.

## 5. Conclusions

The present study revealed a molecular disease signature of TAM/STRMK, and identified abnormal muscle contraction and relaxation kinetics as well as constitutive reticular stress leading to myofiber degeneration as the main cellular pathologies underlying the functional and structural muscle anomalies in *Stim1^R304W/+^* mice. The partial overlap with other diseases including malignant hyperthermia, central core disease, and Duchenne muscular dystrophy points to common pathomechanisms and suggests that a unique therapy may efficiently improve the muscular phenotype in different Ca^2+^-related disorders.

## Data Availability

The authors confirm that the data supporting the findings of this study are available within the article and its Supplementary Materials. RNA-sequencing data were deposited in NCBI GEO: GSE179460.

## Supplementary Materials

The following are available online at https://www.mdpi.com/article/10.3390/cells10071730/s1, Figure S1: Enrichment of immune-related GO terms, Figure S2: Reduced expression of SERCA1 in *Stim1^R304W/+^* tibialis anterior, Figure S3: Decrease of mitochondrial markers in *Stim1^R304W/+^* tibialis anterior, Figure S4: Increased proportion of apoptotic and regenerating fibers in *Stim1^R304W/+^* tibialis anterior, Figure S5: Decreased SERCA1 levels in *Stim1^R304W/+^* soleus, Figure S6: Decreased mitochondrial markers in *Stim1^R304W/+^* soleus, Figure S7: Increased proportion of type I muscle fibers, apoptosis, and regeneration in *Stim1^R304W/+^* soleus, Table S1: List of primers and associated sequences used for qPCR and RT-qPCR.

## References

[1] Berridge M.J., Bootman M.D., Roderick H.L. (2003). Calcium signalling: Dynamics, homeostasis and remodelling. Nat. Rev. Mol. Cell Biol..

[2] Gattineni J. (2014). Inherited disorders of calcium and phosphate metabolism. Curr. Opin. Pediatr..

[3] Chevessier F., Bauche-Godard S., Leroy J.P., Koenig J., Paturneau-Jouas M., Eymard B., Hantai D., Verdiere-Sahuque M. (2005). The origin of tubular aggregates in human myopathies. J. Pathol..

[4] Bohm J., Laporte J. (2018). Gain-of-function mutations in STIM1 and ORAI1 causing tubular aggregate myopathy and Stormorken syndrome. Cell Calcium.

[5] Morin G., Biancalana V., Echaniz-Laguna A., Noury J.B., Lornage X., Moggio M., Ripolone M., Violano R., Marcorelles P., Marechal D. (2020). Tubular aggregate myopathy and Stormorken syndrome: Mutation spectrum and genotype/phenotype correlation. Hum. Mutat..

[6] Lacruz R.S., Feske S. (2015). Diseases caused by mutations in ORAI1 and STIM1. Ann. N. Y. Acad. Sci..

[7] Silva-Rojas R., Laporte J., Bohm J. (2020). STIM1/ORAI1 Loss-of-Function and Gain-of-Function Mutations Inversely Impact on SOCE and Calcium Homeostasis and Cause Multi-Systemic Mirror Diseases. Front. Physiol..

[8] Barone V., Del Re V., Gamberucci A., Polverino V., Galli L., Rossi D., Costanzi E., Toniolo L., Berti G., Malandrini A. (2017). Identification and characterization of three novel mutations in the CASQ1 gene in four patients with tubular aggregate myopathy. Hum. Mutat..

[9] Bohm J., Chevessier F., Maues De Paula A., Koch C., Attarian S., Feger C., Hantai D., Laforet P., Ghorab K., Vallat J.M. (2013). Constitutive activation of the calcium sensor STIM1 causes tubular-aggregate myopathy. Am. J. Hum. Genet..

[10] Bohm J., Lornage X., Chevessier F., Birck C., Zanotti S., Cudia P., Bulla M., Granger F., Bui M.T., Sartori M. (2018). CASQ1 mutations impair calsequestrin polymerization and cause tubular aggregate myopathy. Acta Neuropathol..

[11] Nesin V., Wiley G., Kousi M., Ong E.C., Lehmann T., Nicholl D.J., Suri M., Shahrizaila N., Katsanis N., Gaffney P.M. (2014). Activating mutations in STIM1 and ORAI1 cause overlapping syndromes of tubular myopathy and congenital miosis. Proc. Natl. Acad. Sci. USA.

[12] Stathopulos P.B., Zheng L., Li G.Y., Plevin M.J., Ikura M. (2008). Structural and mechanistic insights into STIM1-mediated initiation of store-operated calcium entry. Cell.

[13] Bohm J., Bulla M., Urquhart J.E., Malfatti E., Williams S.G., O’Sullivan J., Szlauer A., Koch C., Baranello G., Mora M. (2017). ORAI1 Mutations with Distinct Channel Gating Defects in Tubular Aggregate Myopathy. Hum. Mutat..

[14] Endo Y., Noguchi S., Hara Y., Hayashi Y.K., Motomura K., Miyatake S., Murakami N., Tanaka S., Yamashita S., Kizu R. (2015). Dominant mutations in ORAI1 cause tubular aggregate myopathy with hypocalcemia via constitutive activation of store-operated Ca^2+^ channels. Hum. Mol. Genet..

[15] Harris E., Burki U., Marini-Bettolo C., Neri M., Scotton C., Hudson J., Bertoli M., Evangelista T., Vroling B., Polvikoski T. (2017). Complex phenotypes associated with STIM1 mutations in both coiled coil and EF-hand domains. Neuromuscul. Disord..

[16] Misceo D., Holmgren A., Louch W.E., Holme P.A., Mizobuchi M., Morales R.J., De Paula A.M., Stray-Pedersen A., Lyle R., Dalhus B. (2014). A dominant STIM1 mutation causes Stormorken syndrome. Hum. Mutat..

[17] Morin G., Bruechle N.O., Singh A.R., Knopp C., Jedraszak G., Elbracht M., Bremond-Gignac D., Hartmann K., Sevestre H., Deutz P. (2014). Gain-of-Function Mutation in STIM1 (P.R304W) Is Associated with Stormorken Syndrome. Hum. Mutat..

[18] Noury J.B., Bohm J., Peche G.A., Guyant-Marechal L., Bedat-Millet A.L., Chiche L., Carlier R.Y., Malfatti E., Romero N.B., Stojkovic T. (2017). Tubular aggregate myopathy with features of Stormorken disease due to a new STIM1 mutation. Neuromuscul. Disord..

[19] Garibaldi M., Fattori F., Riva B., Labasse C., Brochier G., Ottaviani P., Sacconi S., Vizzaccaro E., Laschena F., Romero N.B. (2017). A novel gain-of-function mutation in ORAI1 causes late-onset tubular aggregate myopathy and congenital miosis. Clin. Genet..

[20] Silva-Rojas R., Treves S., Jacobs H., Kessler P., Messaddeq N., Laporte J., Bohm J. (2019). STIM1 over-activation generates a multi-systemic phenotype affecting the skeletal muscle, spleen, eye, skin, bones and immune system in mice. Hum. Mol. Genet..

[21] Dobin A., Davis C.A., Schlesinger F., Drenkow J., Zaleski C., Jha S., Batut P., Chaisson M., Gingeras T.R. (2013). STAR: Ultrafast universal RNA-seq aligner. Bioinformatics.

[22] Anders S., Pyl P.T., Huber W. (2015). HTSeq—A Python framework to work with high-throughput sequencing data. Bioinformatics.

[23] Love M.I., Huber W., Anders S. (2014). Moderated estimation of fold change and dispersion for RNA-seq data with DESeq2. Genome Biol..

[24] Yu G., Wang L.G., Han Y., He Q.Y. (2012). clusterProfiler: An R package for comparing biological themes among gene clusters. OMICS.

[25] Mayeuf-Louchart A., Hardy D., Thorel Q., Roux P., Gueniot L., Briand D., Mazeraud A., Bougle A., Shorte S.L., Staels B. (2018). MuscleJ: A high-content analysis method to study skeletal muscle with a new Fiji tool. Skelet. Muscle.

[26] Duteil D., Chambon C., Ali F., Malivindi R., Zoll J., Kato S., Geny B., Chambon P., Metzger D. (2010). The transcriptional coregulators TIF2 and SRC-1 regulate energy homeostasis by modulating mitochondrial respiration in skeletal muscles. Cell Metab..

[27] Mansour Z., Bouitbir J., Charles A.L., Talha S., Kindo M., Pottecher J., Zoll J., Geny B. (2012). Remote and local ischemic preconditioning equivalently protects rat skeletal muscle mitochondrial function during experimental aortic cross-clamping. J. Vasc. Surg..

[28] Sciorati C., Rigamonti E., Manfredi A.A., Rovere-Querini P. (2016). Cell death, clearance and immunity in the skeletal muscle. Cell Death Differ..

[29] Schneider M.F., Chandler W.K. (1973). Voltage dependent charge movement of skeletal muscle: A possible step in excitation-contraction coupling. Nature.

[30] Lamb G.D., Junankar P.R., Stephenson D.G. (1995). Raised intracellular [Ca2+] abolishes excitation-contraction coupling in skeletal muscle fibres of rat and toad. J. Physiol..

[31] Murphy R.M., Dutka T.L., Horvath D., Bell J.R., Delbridge L.M., Lamb G.D. (2013). Ca2+-dependent proteolysis of junctophilin-1 and junctophilin-2 in skeletal and cardiac muscle. J. Physiol..

[32] Deluca H.F., Engstrom G.W. (1961). Calcium uptake by rat kidney mitochondria. Proc. Natl. Acad. Sci. USA.

[33] Rizzuto R., De Stefani D., Raffaello A., Mammucari C. (2012). Mitochondria as sensors and regulators of calcium signalling. Nat. Rev. Mol. Cell Biol..

[34] Rossi A.E., Dirksen R.T. (2006). Sarcoplasmic reticulum: The dynamic calcium governor of muscle. Muscle Nerve.

[35] Bahar E., Kim H., Yoon H. (2016). ER Stress-Mediated Signaling: Action Potential and Ca^2+^ as Key Players. Int. J. Mol. Sci..

[36] Pullen A.H. (1977). The distribution and relative sized of fibre types in the extensor digitorum longus and soleus muscles of the adult rat. J. Anat..

[37] Pullen A.H. (1977). The distribution and relative sizes of three histochemical fibre types in the rat tibialis anterior muscle. J. Anat..

[38] Lamboley C.R., Murphy R.M., McKenna M.J., Lamb G.D. (2013). Endogenous and maximal sarcoplasmic reticulum calcium content and calsequestrin expression in type I and type II human skeletal muscle fibres. J. Physiol..

[39] Fraysse B., Desaphy J.F., Pierno S., De Luca A., Liantonio A., Mitolo C.I., Camerino D.C. (2003). Decrease in resting calcium and calcium entry associated with slow-to-fast transition in unloaded rat soleus muscle. FASEB J..

[40] Parekh A.B., Penner R. (1997). Store depletion and calcium influx. Physiol. Rev..

[41] Sultana N., Dienes B., Benedetti A., Tuluc P., Szentesi P., Sztretye M., Rainer J., Hess M.W., Schwarzer C., Obermair G.J. (2016). Restricting calcium currents is required for correct fiber type specification in skeletal muscle. Development.

[42] Maier A., Gorza L., Schiaffino S., Pette D. (1988). A combined histochemical and immunohistochemical study on the dynamics of fast-to-slow fiber transformation in chronically stimulated rabbit muscle. Cell Tissue Res..

[43] Jungbluth H., Sewry C.A., Muntoni F. (2011). Core myopathies. Semin. Pediatr. Neurol..

[44] Avila G., Dirksen R.T. (2001). Functional effects of central core disease mutations in the cytoplasmic region of the skeletal muscle ryanodine receptor. J. Gen. Physiol..

[45] Dirksen R.T., Avila G. (2002). Altered ryanodine receptor function in central core disease: Leaky or uncoupled Ca^2+^ release channels?. Trends Cardiovasc. Med..

[46] Kraeva N., Zvaritch E., Rossi A.E., Goonasekera S.A., Zaid H., Frodis W., Kraev A., Dirksen R.T., Maclennan D.H., Riazi S. (2013). Novel excitation-contraction uncoupled RYR1 mutations in patients with central core disease. Neuromuscul. Disord..

[47] Schartner V., Laporte J., Bohm J. (2019). Abnormal Excitation-Contraction Coupling and Calcium Homeostasis in Myopathies and Cardiomyopathies. J. Neuromuscul. Dis..

[48] Conte E., Pannunzio A., Imbrici P., Camerino G.M., Maggi L., Mora M., Gibertini S., Cappellari O., De Luca A., Coluccia M. (2021). Gain-of-Function STIM1 L96V Mutation Causes Myogenesis Alteration in Muscle Cells from a Patient Affected by Tubular Aggregate Myopathy. Front. Cell Dev. Biol..

[49] Bohm J., Chevessier F., Koch C., Peche G.A., Mora M., Morandi L., Pasanisi B., Moroni I., Tasca G., Fattori F. (2014). Clinical, histological and genetic characterisation of patients with tubular aggregate myopathy caused by mutations in STIM1. J. Med. Genet..

[50] Manning B.M., Quane K.A., Ording H., Urwyler A., Tegazzin V., Lehane M., O’Halloran J., Hartung E., Giblin L.M., Lynch P.J. (1998). Identification of novel mutations in the ryanodine-receptor gene (RYR1) in malignant hyperthermia: Genotype-phenotype correlation. Am. J. Hum. Genet..

[51] Gillard E.F., Otsu K., Fujii J., Khanna V.K., de Leon S., Derdemezi J., Britt B.A., Duff C.L., Worton R.G., MacLennan D.H. (1991). A substitution of cysteine for arginine 614 in the ryanodine receptor is potentially causative of human malignant hyperthermia. Genomics.

[52] Denborough M.A., Forster J.F., Lovell R.R., Maplestone P.A., Villiers J.D. (1962). Anaesthetic deaths in a family. Br. J. Anaesth..

[53] MacLennan D.H., Phillips M.S. (1992). Malignant hyperthermia. Science.

[54] Eltit J.M., Bannister R.A., Moua O., Altamirano F., Hopkins P.M., Pessah I.N., Molinski T.F., Lopez J.R., Beam K.G., Allen P.D. (2012). Malignant hyperthermia susceptibility arising from altered resting coupling between the skeletal muscle L-type Ca2+ channel and the type 1 ryanodine receptor. Proc. Natl. Acad. Sci. USA.

[55] Weiss R.G., O’Connell K.M., Flucher B.E., Allen P.D., Grabner M., Dirksen R.T. (2004). Functional analysis of the R1086H malignant hyperthermia mutation in the DHPR reveals an unexpected influence of the III-IV loop on skeletal muscle EC coupling. Am. J. Physiol. Cell Physiol..

[56] Lavorato M., Gupta P.K., Hopkins P.M., Franzini-Armstrong C. (2016). Skeletal Muscle Microalterations in Patients Carrying Malignant Hyperthermia-Related Mutations of the e-c Coupling Machinery. Eur. J. Transl. Myol..

[57] Cordero-Sanchez C., Riva B., Reano S., Clemente N., Zaggia I., Ruffinatti F.A., Potenzieri A., Pirali T., Raffa S., Sangaletti S. (2019). A luminal EF-hand mutation in STIM1 in mice causes the clinical hallmarks of tubular aggregate myopathy. Dis. Models Mech..

[58] Dainese M., Quarta M., Lyfenko A.D., Paolini C., Canato M., Reggiani C., Dirksen R.T., Protasi F. (2009). Anesthetic- and heat-induced sudden death in calsequestrin-1-knockout mice. FASEB J..

[59] Lee C.S., Hanna A.D., Wang H., Dagnino-Acosta A., Joshi A.D., Knoblauch M., Xia Y., Georgiou D.K., Xu J., Long C. (2017). A chemical chaperone improves muscle function in mice with a RyR1 mutation. Nat. Commun..

[60] Lee K.W., Maeng J.S., Choi J.Y., Lee Y.R., Hwang C.Y., Park S.S., Park H.K., Chung B.H., Lee S.G., Kim Y.S. (2012). Role of Junctin protein interactions in cellular dynamics of calsequestrin polymer upon calcium perturbation. J. Biol. Chem..

[61] Wang L., Zhang L., Li S., Zheng Y., Yan X., Chen M., Wang H., Putney J.W., Luo D. (2015). Retrograde regulation of STIM1-Orai1 interaction and store-operated Ca^2+^ entry by calsequestrin. Sci. Rep..

[62] Park C.Y., Shcheglovitov A., Dolmetsch R. (2010). The CRAC channel activator STIM1 binds and inhibits L-type voltage-gated calcium channels. Science.

[63] Burr A.R., Molkentin J.D. (2015). Genetic evidence in the mouse solidifies the calcium hypothesis of myofiber death in muscular dystrophy. Cell Death Differ..

[64] Kargacin M.E., Kargacin G.J. (1996). The sarcoplasmic reticulum calcium pump is functionally altered in dystrophic muscle. Biochim. Biophys. Acta.

[65] Begam M., Abro V.M., Mueller A.L., Roche J.A. (2016). Sodium 4-phenylbutyrate reduces myofiber damage in a mouse model of Duchenne muscular dystrophy. Appl. Physiol. Nutr. Metab..

[66] De Luca A., Pierno S., Liantonio A., Cetrone M., Camerino C., Simonetti S., Papadia F., Camerino D.C. (2001). Alteration of excitation-contraction coupling mechanism in extensor digitorum longus muscle fibres of dystrophic mdx mouse and potential efficacy of taurine. Br. J. Pharmacol..

[67] Goonasekera S.A., Lam C.K., Millay D.P., Sargent M.A., Hajjar R.J., Kranias E.G., Molkentin J.D. (2011). Mitigation of muscular dystrophy in mice by SERCA overexpression in skeletal muscle. J. Clin. Investig..

[68] Terrill J.R., Pinniger G.J., Graves J.A., Grounds M.D., Arthur P.G. (2016). Increasing taurine intake and taurine synthesis improves skeletal muscle function in the mdx mouse model for Duchenne muscular dystrophy. J. Physiol..

[69] Grosse J., Braun A., Varga-Szabo D., Beyersdorf N., Schneider B., Zeitlmann L., Hanke P., Schropp P., Muhlstedt S., Zorn C. (2007). An EF hand mutation in Stim1 causes premature platelet activation and bleeding in mice. J. Clin. Investig..

[70] Gamage T.H., Gunnes G., Lee R.H., Louch W.E., Holmgren A., Bruton J.D., Lengle E., Kolstad T.R.S., Revold T., Amundsen S.S. (2018). STIM1 R304W causes muscle degeneration and impaired platelet activation in mice. Cell Calcium.

