# Supplementary material for: Pathophysiological Effects of Overactive STIM1 on Murine Muscle Function and Structure
Source: Cells. 2021 Jul 8;10(7):1730. doi: 10.3390/cells10071730 (PMC8304505; doi:10.3390/cells10071730)
Supplement: Supplementary file 1 [file cells-10-01730-s001.zip › cells-1241483-supplementary.pdf]

SUPPLEMENTAL MATERIAL

Supplemental Figure S1. Enrichment of immune-related GO-terms. (A) Classification of the dysregulated genes in *Stim1*$^{R304W/+}$ tibialis anterior into GO terms reveals an important number of groups associated with the immune response (n=4). (B) RNAseq uncovered a total of 3349 differentially expressed genes (DEG) in *Stim1*$^{R304W/+}$ tibialis anterior compared with the WT. Following removal of the immune-related GO terms, 2841 DEG remained.

Supplemental Figure S2. **Reduced expression of SERCA1 in *Stim1*$^{R304W/+}$ tibialis anterior.** (A-C) Western blots showing the SERCA1, DHPR and RyR1 protein levels in WT and *Stim1*$^{R304W/+}$ tibialis anterior (n=6, corresponding to the graph in Fig. 1D and 1F). Ponceau staining served as loading control.

Supplemental Figure S3. **Decrease of mitochondrial markers in *Stim1*$^{R304W/+}$ tibialis anterior.** (A-C) Western blots on muscle extracts showing a decrease of PGC1α protein level (graph in Fig. 2C), and of the mitochondrial electron transport chain proteins ATP5A, UQCRC2, SDHB, and NDUFB8 (n=6). Ponceau staining served as loading control. (D) H_2O_2 production is slightly reduced in *Stim1*$^{R304W/+}$ tibialis anterior muscle fibers. Significant differences are illustrated as *(p<0.05), **(p<0.01), and ***(p<0.001).

Supplemental Figure S4. **Increased proportion of apoptotic and regenerating fibers in *Stim1*$^{R304W/+}$ tibialis anterior**. Immunofluorescence showing apoptotic fibers on *Stim1*$^{R304W/+}$ muscle sections as illustrated by the signal of cleaved caspase-3 (top), and regenerating fibers expressing embryonic myosin (bottom). Wheat germ agglutinin (WGA) outlines the myofibers. Scales correspond to 50 µm.

Supplemental Figure S5. **Decreased SERCA1 levels in *Stim1$^{R304W/+}$* soleus.** (A-C) Western blots showing the SERCA1, DHPR and RyR1 levels in muscle extracts (n=4-5, graph in Fig. 5B). The cross indicates an incorrectly charged lane removed from the analysis. Ponceau staining serves as loading control.

Supplemental Figure S6. **Decreased mitochondrial markers in *Stim1$^{R304W/+}$* soleus**. (A-C) Western blots revealing reduced levels of PGC1α (graph in Fig. 5C) and of ATP5A, UQCRC2, SDHB, and NDUFB8, representing proteins of the mitochondrial electron transport chain complexes V, III, II, and I in *Stim1$^{R304W/+}$* muscle samples compared with WT. Ponceau staining served as loading control, and crosses indicate incorrectly loaded lanes removed from the analysis. (D) Decreased H_2O_2 production in *Stim1$^{R304W/+}$* soleus. Significant differences are illustrated as *($p<0.05$).

Supplemental Figure S7. **Increased proportion of type I muscle fibers, apoptosis, and regeneration in *Stim1$^{R304W/+}$* soleus.** (A) Representative muscle cross sections and statistical analysis showing the fiber type pattern and highlighting an increased proportion of type I fibers in *Stim1$^{R304W/+}$* soleus compared with the control (n=4-5). Type I fibers appear in red, intermediate type IIa fibers in green, and fast type IIb fibers in blue. The remaining fibers are fast IIx. Significant differences are illustrated as **** ($p<0.0001$). (B) Apoptotic fibers in *Stim1$^{R304W/+}$* soleus staining positive for cleaved caspase-3 (top), and regenerating fibers expressing embryonic myosin (bottom). Wheat germ agglutinin (WGA) outlines the myofibers.

Supplemental Table S1. List of primers and associated sequences used for qPCR and RT-qPCR.

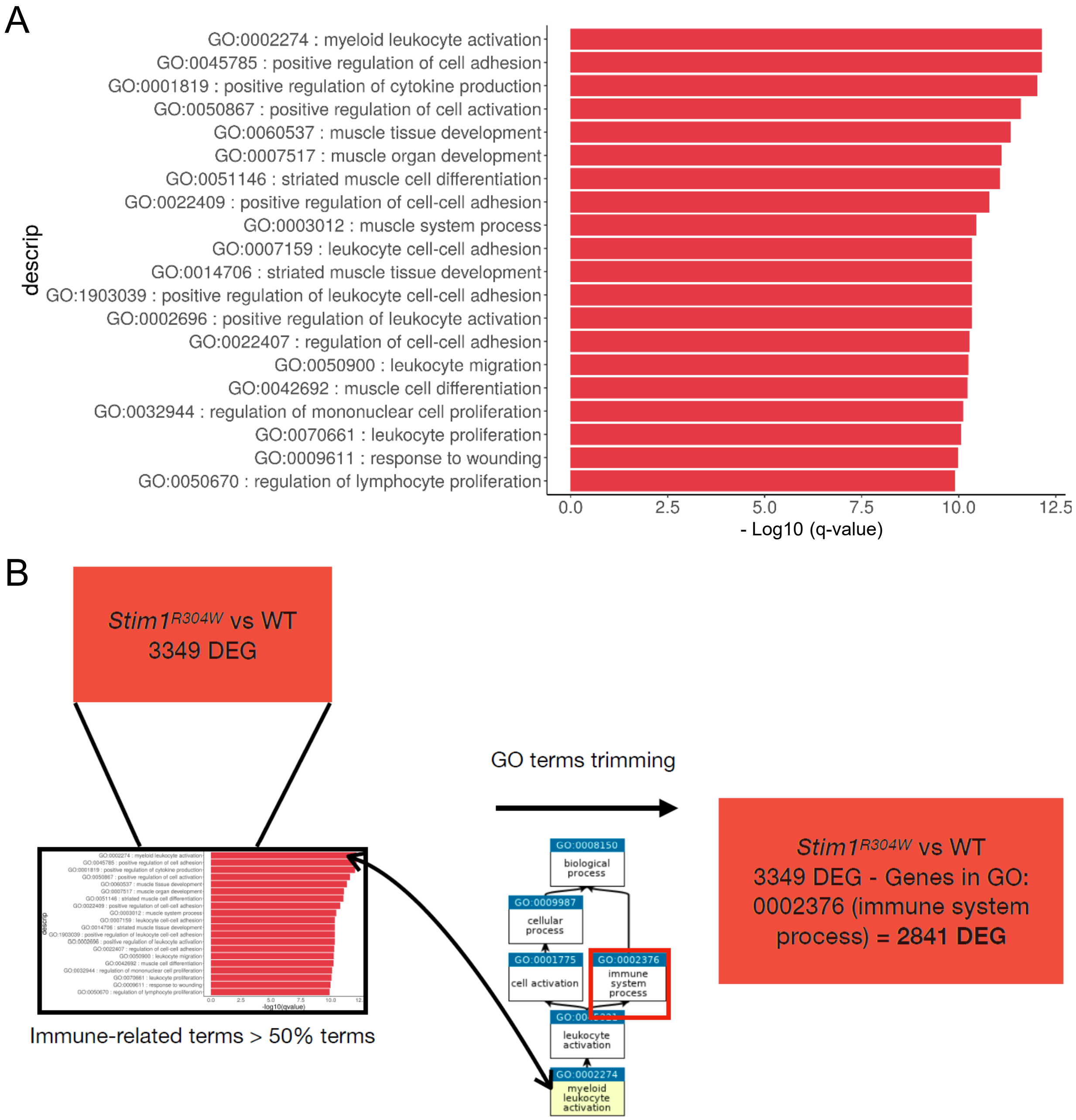

Supplemental Figure S1

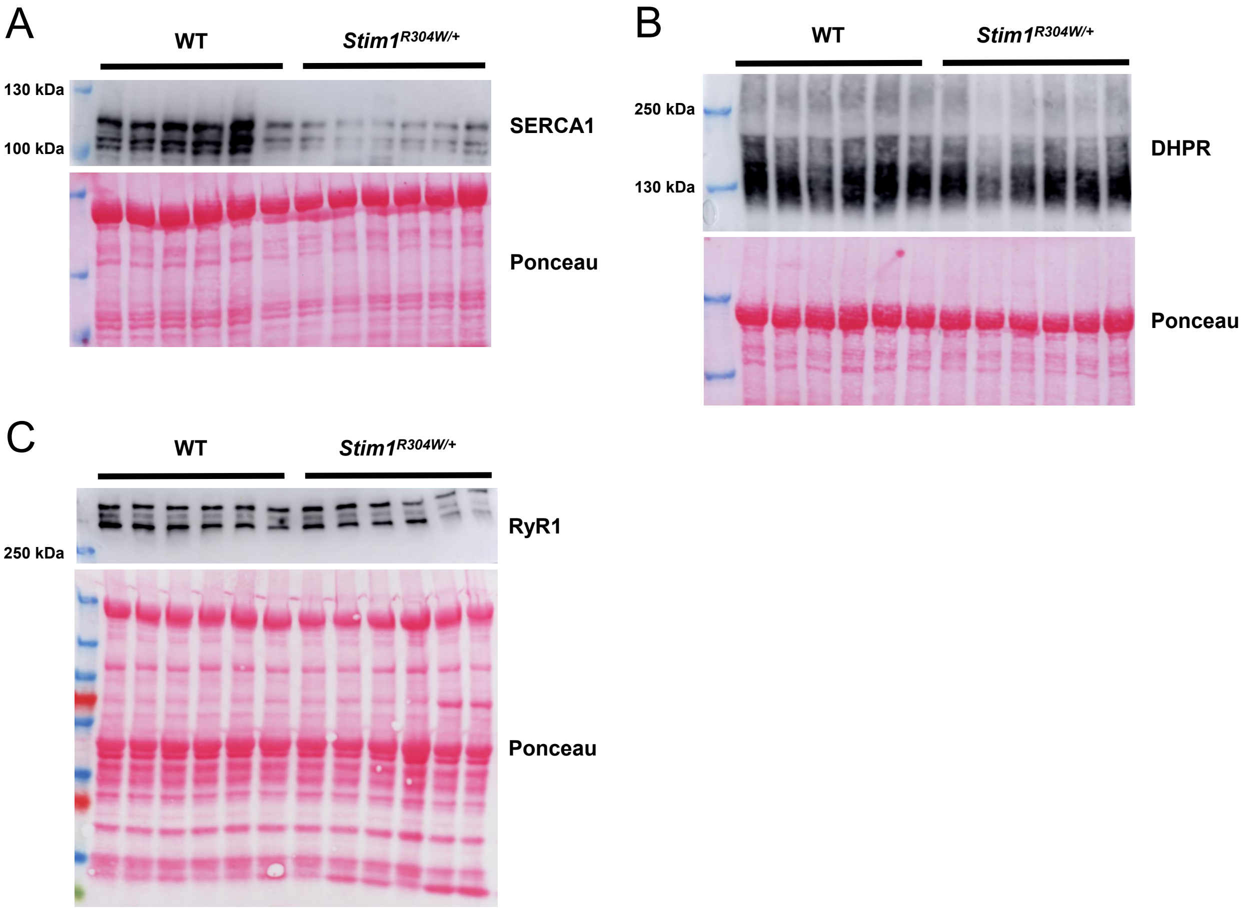

Supplemental Figure S2

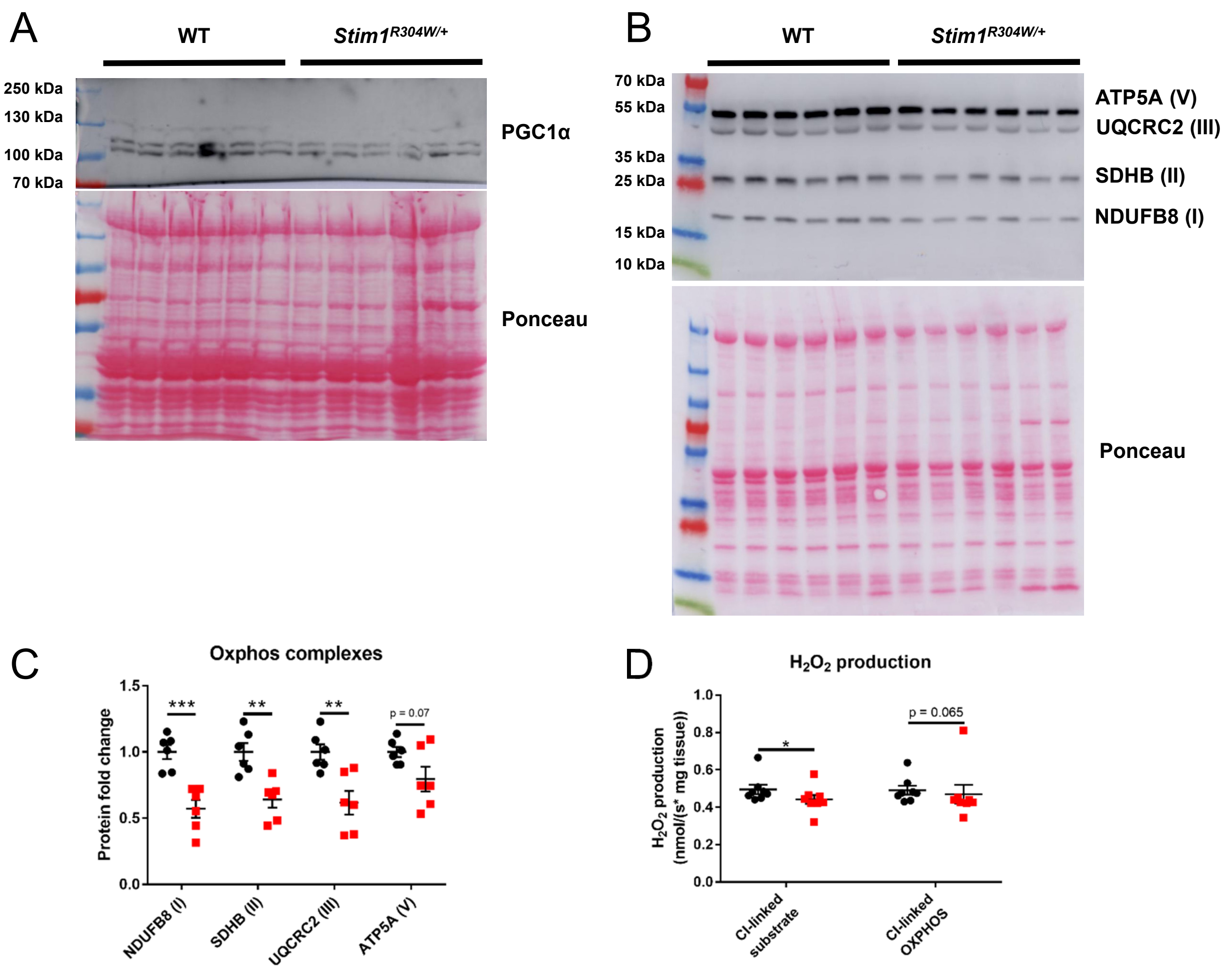

Supplemental Figure S3

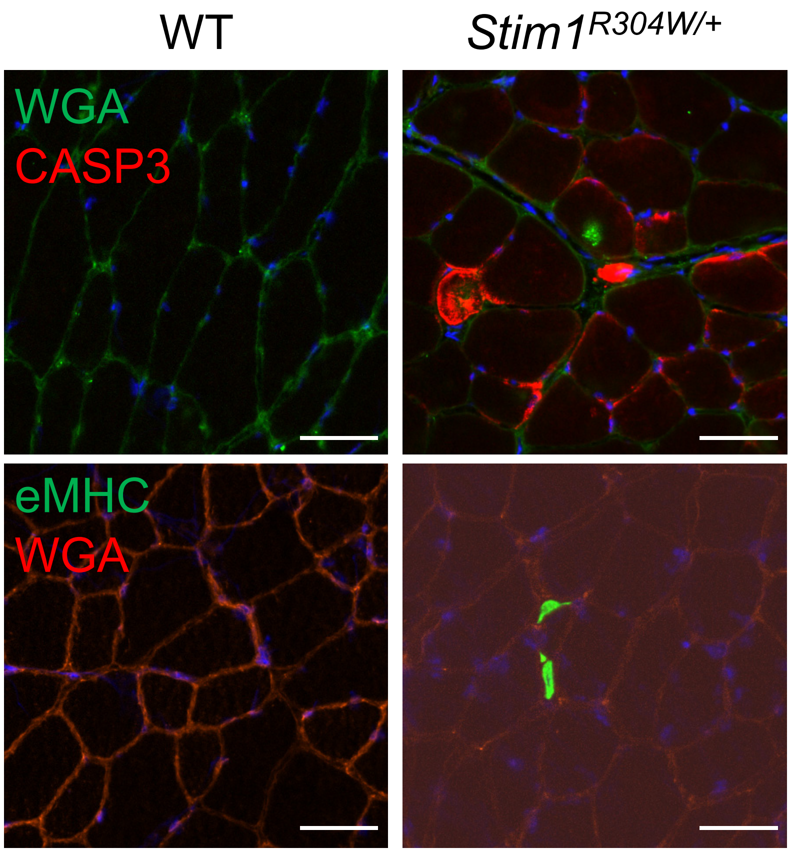

Supplemental Figure S4

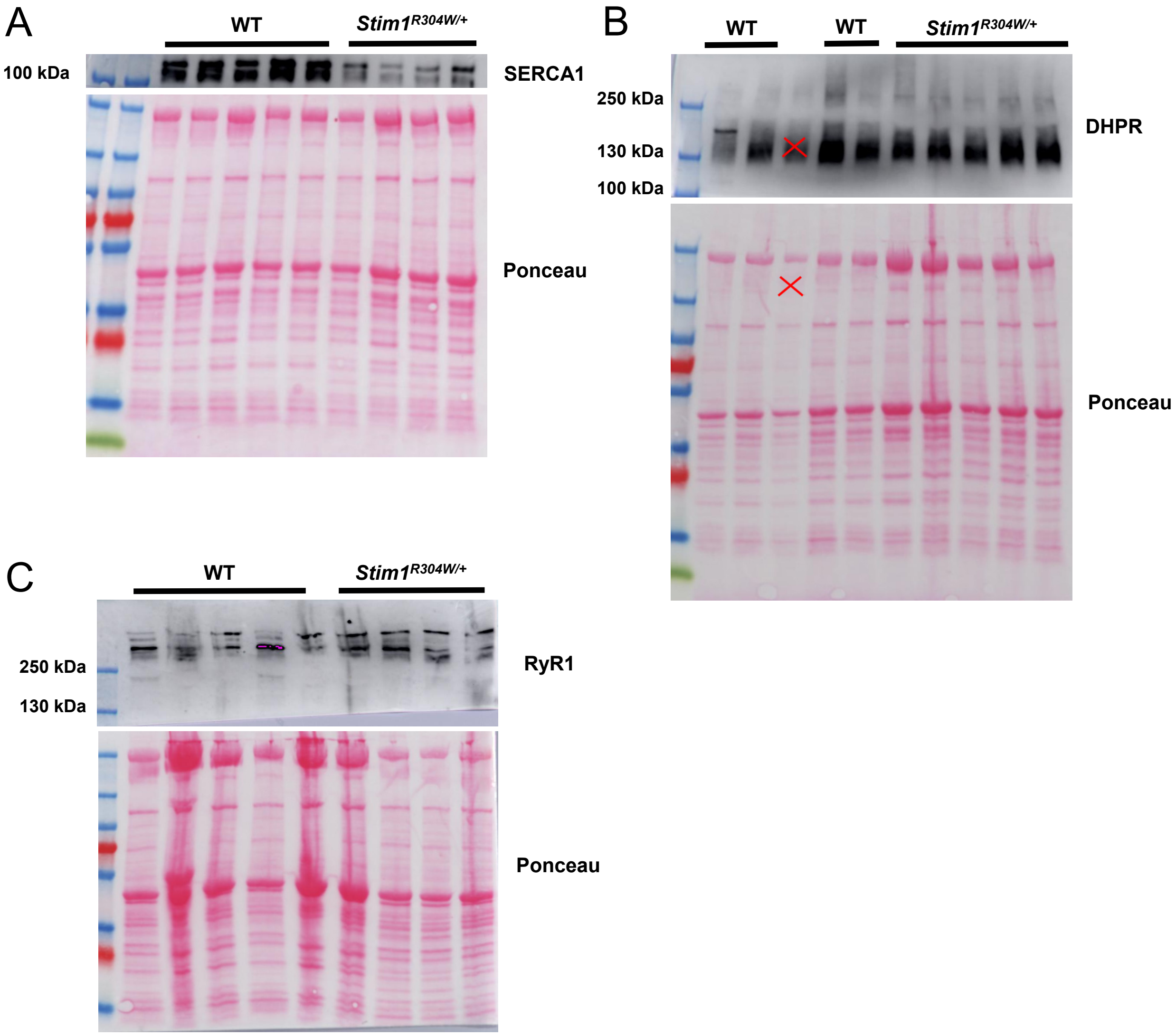

Supplemental Figure S5

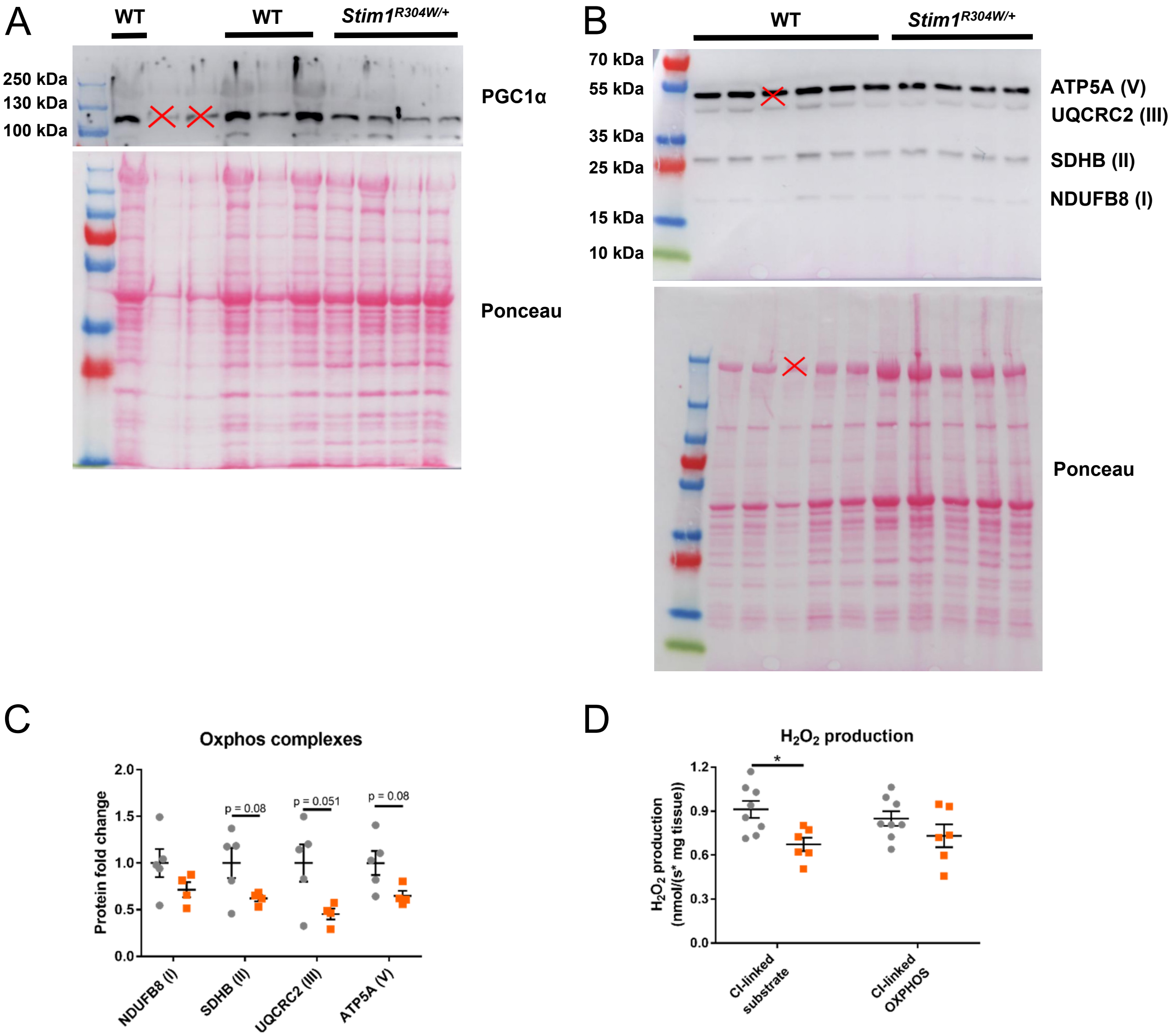

Supplemental Figure S6

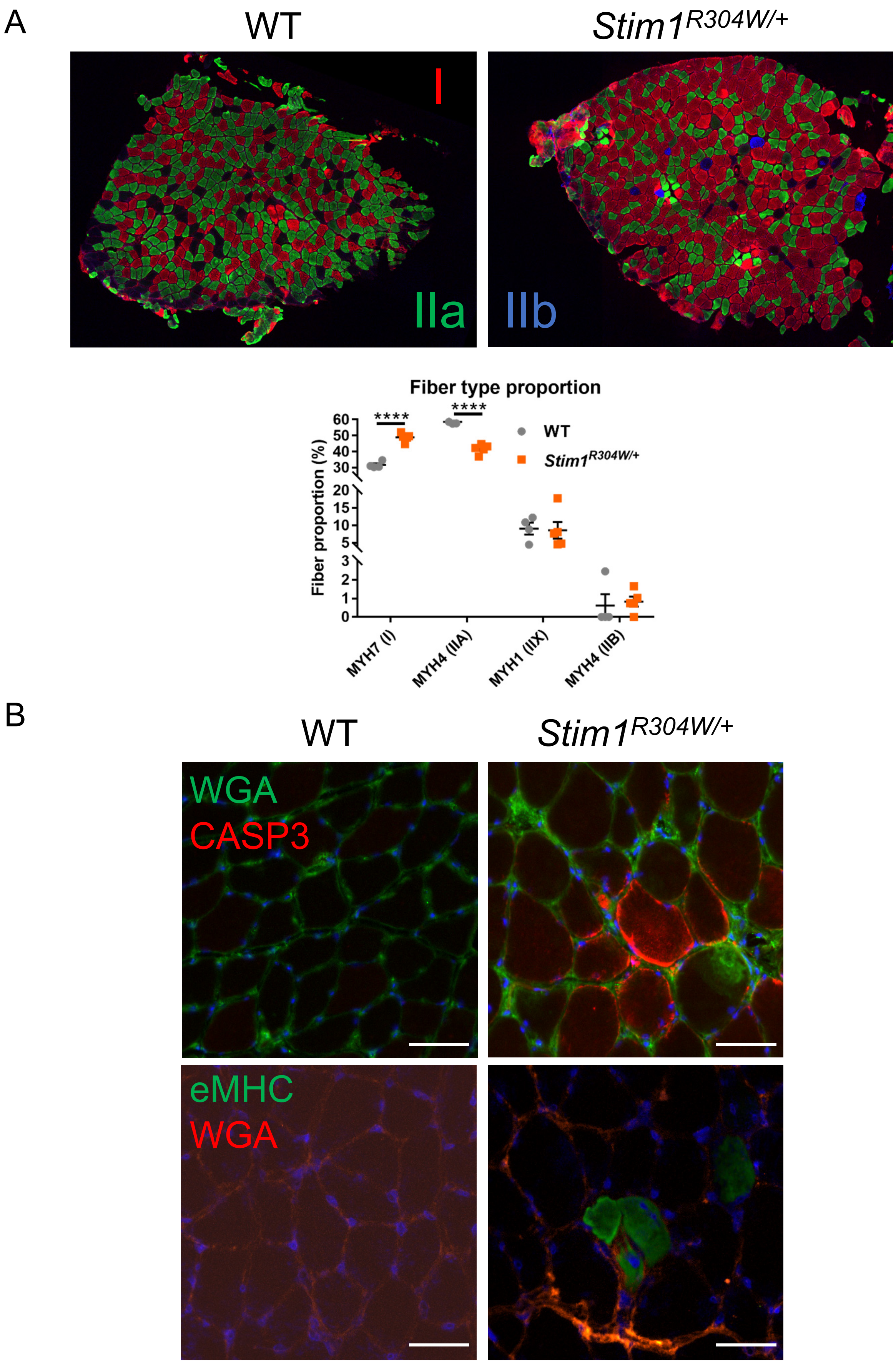

Supplemental Figure S7

Supplemental Table S1.

Pathway	Gene	Forward primer	Reverse primer
Ca^{2+} extrusion	*Atp2b1*	TTATCAACCTCCGGAAGGGGATAAT	GCTCCTTCAATCCACCCCGTTTCT
	Slc8a1	GTTTGTTGCTCTTGGAACCTCGGTG	GTGACATTGCCTATAGACGCATCTG
	Slc8a3	TTTTGTGGCATTCGGCACCTCTGTG	GTGACGTTGCCAATGGAAGCATCTG
SR refilling	*Atp2a1*	CCCTCACCACCAACCAGATGTCAGTT	CAGTGATGGAGAACTCGTTCAGTGAG C
	Sln	TGTCCTCATCACCGTTCTCC	TGGAGTATAGCATGGCCCCT
	Plb	AGTGCAATACCTCACTCGCT	TTCTGACGTGCTTGCTGAGG
EC coupling	*Cana1s*	AACCTGGTGCTGGGTGTCCTG	TCTCTCGGAGCTTTTGGAAGGTTC
	Ryr1	CAGTGGACTACCTCCTGCGGC	GTTTCTCTTTCCCTGTTCCTCGATG
Mitochondrial biogenesis	*Ppargc1a*	GCAGGTCGAACGAAACTGAC	CTTGCTCTTGGTGGAAGCAG
	Sirt1	GGCCGCGGATAGGTCCA	AACAATCTGCCACAGCGTCA
	Nrf1	ATGTCCGCACAGAAGAGCAA	TGTACCAACCTGGATGAGCG
	Tfam	ATAGGCACCGTATTGCGTGA	AGTTTTGCATCTGGGTGTTTAGC
mtDNA copy number	*mt16S*	CTAGAAACCCCGAAACCAAA	CCAGCTATCACCAAGCTCGT
	Cox2	AATTAGCTCCTTAGTCCTCT	CTTGGTCGGTTTGATGTTAC
	Loop	GCGTTATCGCCTCATACGTT	GATTGGGTTTTGCGGACTAA
Mitochondrial transport	*Rhot1*	GGCCATGTACCCGCACG	ATGTGTTTTGGTAGGCCGGT
	Trak1	GTCTCCAGACATCACCCACC	TATCGAGGACCACGTTGCTG
Mitochondrial dynamics	*Dnm1l*	GAGTTGAAGCAGAAGAATGGGG	CGCCTACAGGTACTTTGGTCA
	Fis1	GCAACTACCGGCTCAAGGAAT	GTGAGGCTGCCTTCAGGATT
	Opa1	TGAGGCCCTTCTCTTGTTAGG	TCTTTGTCTGACACCTTCCTGT
	Mfn2	GCTAGAAACTTCTCCTCTGTTCCA	CTTGACGGTGACGATGGAGT
Unfolded protein response	*Hspa5*	CTATTCCTGCGTCGGTGTGT	ATTCCAAGTGCGTCCGATGA
	Hsp90b1	CCACTCAAATCGAACACGGC	AGATTCCGCCTCCTTTCTGC
	Xbp1	AGAAGAGAACCACAAACTCCAGC	ACATAGTCTGAGTGCTGCGG
	Ddit3	CCAGAATAACAGCCGGAACC	ATCCTCATACCAGGCTTCCA
Muscle regeneration	*Myh3*	CTTCACCTCTAGCCGGAATGGT	AATTGTCAGGAGCCACGAAAAT
	Myh8	CAGGAGCAGGAATGATGCTCTGAG	AGTTCCTCAAACTTTCAGCAGCCAA
RT-qPCR control	*Rpl27*	AAGCCGTCATCGTGAAGAACA	CTTGATCTTGGATCGCTTGGC
qPCR control	*B2M*	ATGGGAAGCCGAACATACTG	CAGTCTCAGTGGGGGTGAAT
